# FAM72A promotes glioma progression by regulating mitophagy through the Pink1/Parkin signaling pathway

**DOI:** 10.7150/jca.82949

**Published:** 2023-04-02

**Authors:** Yibin Zeng, Cui Xiong, Nan Tang, Siqi Wang, Zhiyong Xiong, Tao Liang, Qiangping Wang, Menglong Li, Junjun Li

**Affiliations:** 1 Department of Neurosurgery, Union Hospital, Tongji Medical College, Huazhong University of Science and Technology, 1277 Jiefang Avenue, Wuhan, Hubei, 430022, China.; 2 Department of Endocrinology, Union Hospital, Tongji Medical College, Huazhong University of Science and Technology, 1277 Jiefang Avenue, Wuhan, Hubei, 430022, China.; 3 Department of Radiology, Union Hospital, Tongji Medical College, Huazhong University of Science and Technology, 1277 Jiefang Avenue, Wuhan, Hubei, 430022, China.; 4 Department of Clinical Laboratory, Union Hospital, Tongji Medical College, Huazhong University of Science and Technology, 1277 Jiefang Avenue, Wuhan, Hubei, 430022, China.; 5 Department of Neurosurgery, Nanshi Hospital of Nanyang, Henan University, No. 988, Zhongzhou West Road, Nanyang City, 442000, China.

**Keywords:** FAM72A, Parkin/Pink1, PD-L1, mitophagy, glioma

## Abstract

**Background:** There is growing evidence that aberrant expression of FAM72A contributes to biological dysfunction, especially mitochondrial dysfunction. However, its role in most tumors remains unclear, especially in glioma.

**Methods:** Herein, a high-throughput sequencing approach was used here to identify FAM72A as the target molecule. Next, we detected the protein and mRNA expression levels of FAM72A in normal brain tissue (NBT) as well as different grades of glioma tissue. CCK-8, colony formation, Transwell assays, and Western blotting, were all used to determine the molecular effects of FAM72A on glioma cells.

**Results:** FAM72A was significantly upregulated in glioma, was significantly correlated with WHO grade and was associated with poor clinical outcomes. In functional assays, FAM72A was shown to promote glioma cell growth. Subsequent mechanistic studies indicated that FAM72A promoted glioma progression by regulating mitophagy through the Pink1/Parkin signaling pathway. In addition, FAM72A promoted mitophagy and maintained Pink1 stability through the Pink1/Parkin signaling pathway. Finally, FAM72A promoted tumor immune escape by upregulating PD-L1 expression.

**Conclusion:** All of these data indicate that FAM72A confers an aggressive phenotype and poor prognosis on gliomas. Targeting FAM72A might represent a new therapeutic strategy for glioma.

## Introduction

Glioma is a highly malignant tumor, especially glioblastoma (GBM). Despite continuous improvement in comprehensive treatment methods such as surgery, radiotherapy, chemotherapy and TTF, the survival time of patients with GBM has only increased from 12.1 to 14.6 months 1. Thus, finding novel biomarkers is crucial to providing patients with gliomas with valid and reliable survival predictions as well as more aggressive treatment.

Mitochondria serve as centers of metabolic and signal transduction, as well as cellular signal transduction, and regulate cell signal transduction and biological functions 2. When external stimuli cause changes in the surrounding environment of cells, mitochondria regulate their shape and function through dynamic changes in fission and fusion, in turn causing changes in cell biological functions 3, 4. If pathological factors continue to be stimulated, an imbalance of mitochondrial fission/fusion occurs, resulting in the disturbance of mitochondrial function and cell signal transduction, further leading to abnormal biological functions such as cell differentiation, proliferation, and apoptosis and thereby causing cardiovascular, diabetes, and tumor diseases 5. Accordingly, mitochondrial fission/fusion homeostasis is an important mechanism for regulating cell signal transduction and biological functions. Furthermore, mitophagy is critical for maintaining mitochondrial fission and fusion homeostasis by selectively removing damaged mitochondria 6, 7. The Pink1/Parkin pathway is a common mitophagy pathway 8, 9. Pink1 is located in the inner mitochondrial membrane and is recognized and cleaved by the mitochondrial protease PARL after entering the inner mitochondrial membrane. Thereafter, Pink1 is degraded by the proteasome in normal mitochondria 10, 11. Parkin promotes mitophagy in damaged mitochondria by stabilizing Pink1 on the outer membrane 12.

FAM72A, also known as Ugene, is located on chromosome 1q32.1, its encoded protein includes 149 amino acids, its expression is increased in colon cancer, breast cancer, lung cancer, uterine and ovarian tumors, and it functions by binding to DNA-glycosylase 2 (UNG2) of the base excision repair (BER) pathway 13. Overexpression of FAM17A reduces ROS production and alleviates H_2_O_2_-induced loss of mitochondrial membrane potential in nasopharyngeal carcinoma cells, thereby promoting tumor cell survival 14. Two back-to-back articles published in Nature in 2021 also confirmed the important role of FAM72A in living organisms 15, 16. The authors screened for class-switch recombination during B cell maturation by genome-wide CRISPR-Cas9 knockout CSR-related genes and found that deletion of FAM72A resulted in CSR deficiency in B cells 15. FAM72A also promotes error-prone repair of DNA, and cells overexpressing FAM72A have higher rates of gene mutation 16. However, neither FAM72A nor its function in glioma has yet been reported, and whether FAM72A contributes to tumor progression through Pink1/Parkin signaling remains unknown.

In this study, glioma was demonstrated to have higher FAM72A expression and distinct mitochondrial morphology. FAM72A mediates glioma mitochondrial fission, induces mitophagy, and promotes glioma progression via mitophagy. Mechanistically, FAM72A promoted mitophagy and maintained the stability of Pink1 through Pink1/Parkin. Finally, FAM72A was found to promote the immune escape of glioma by upregulating PD-L1 expression.

## Methods

### Clinical samples

Surgical specimens from patients with glioma were collected from Wuhan Union Hospital (Wuhan, China). Informed consent was provided by all patients. Details on patient characteristics are provided in Supplementary Tables 1 and 2. This study was performed in accordance with the guidelines in the Declaration of Helsinki, and all clinical samples and clinical information were gathered and handled in compliance with relevant guidelines and regulations. Further details are provided in the Supplementary Material.

### Cell culture and treatment

The protocol described in previous publications 17, 18 was employed for cell culture. All cells, frequently screened for mycoplasma contamination, were subjected to short tandem repeat analysis. Cells were treated with 8 μM Mdivi-1 for 24 h. In the Supplemental Material, a detailed description of the procedure is provided.

### Plasmids, small interfering RNA (siRNA), and transfection

GeneChem Co. Ltd. (Shanghai, China) provided all siRNAs (Supplementary Table 3). All protocols were based on the manufacturer's instructions. The Supplemental Material provides detailed instructions.

### Western blotting (WB)

The related protocol details were published in previous studies 19-21. The details of all antibodies are listed in Supplementary Table 4.

### Real-time quantitative RT-PCR (qRT-PCR)

The assay was performed according to the manufacturer's instructions. The 2^-ΔΔCt^ method was used to normalize the expression data to that of GAPDH, which served as a control 22. GeneCreate (Wuhan, China) synthesized the primers used in this study. Primer sequences are given in Supplementary Table 5. Detailed instructions are provided in the Supplementary Material.

### Bioinformatics analysis

We downloaded all datasets from The Cancer Genome Atlas (TCGA) (https://cancergenome.nih.gov/) and Genotype Tissue Expression (GTEx) 23. Independent sample t-tests comparing the two groups are represented as t-tests. A detailed description of the methodology is provided in the Supplementary Material.

### Confocal Microscopy

HBAD-EGFP-LC3 and HBAD-h-mito-dsRed adenoviral particles were purchased from HanBio (Shanghai, China). After infection with adenoviral particles, the cells were cultured for another 24 h. Subsequently, glioma cells were washed three with PBS before incubation with 4% paraformaldehyde at 37 °C for 1 h in the dark. The sections were then covered with VECTASHIELD^®^ Antifade Mounting Medium containing DAPI (H-1200, Vector Laboratories, Inc., Burlingame, CA, USA). Finally, the samples were imaged using a Nikon A1+/A1R+ confocal laser microscope (Nikon, Tokyo, Japan).

### Transmission electron microscopy (TEM) and immunohistochemistry (IHC)

These assays were performed as described in a previous publication 18, 24, 25 and in the Supplementary Materials.

### Brain orthotropic xenografts

The study used female BALB/c nude mice that were 6-8 weeks old. Stereotactically injected U-87MG and U-251 cells were resuspended in cold PBS. Bioluminescence imaging was conducted with intraperitoneal injection of D-luciferin (16 mg/mL, 180 μL), and live animal imaging was conducted using the IVIS imaging system. The animal experiments were performed as described in previous publications 19, 20, 26.

### Statistical analysis

Statistical analysis was performed using GraphPad Prism 8.0 (GraphPad Inc., La Jolla, CA, USA), and statistical descriptions are presented as the mean ± SD or median (interquartile range). According to whether the data obey the normal distribution and the homogeneity of variance, the comparison between the two groups was carried out using a t-test, t' test or rank sum test. One-way ANOVA, Brown-Forsythe ANOVA, or rank sum test were used to compare overall means among multiple groups. Pairwise comparisons were performed using Dunnett, Dunnett T3, or Dunn's method for P value correction to control for the overall probability of type I error; two-sided P<0.05 was defined as a statistically significant difference.

## Results

### The overexpression of FAM72A in human gliomas is associated with a poor prognosis

**Figure 1:**
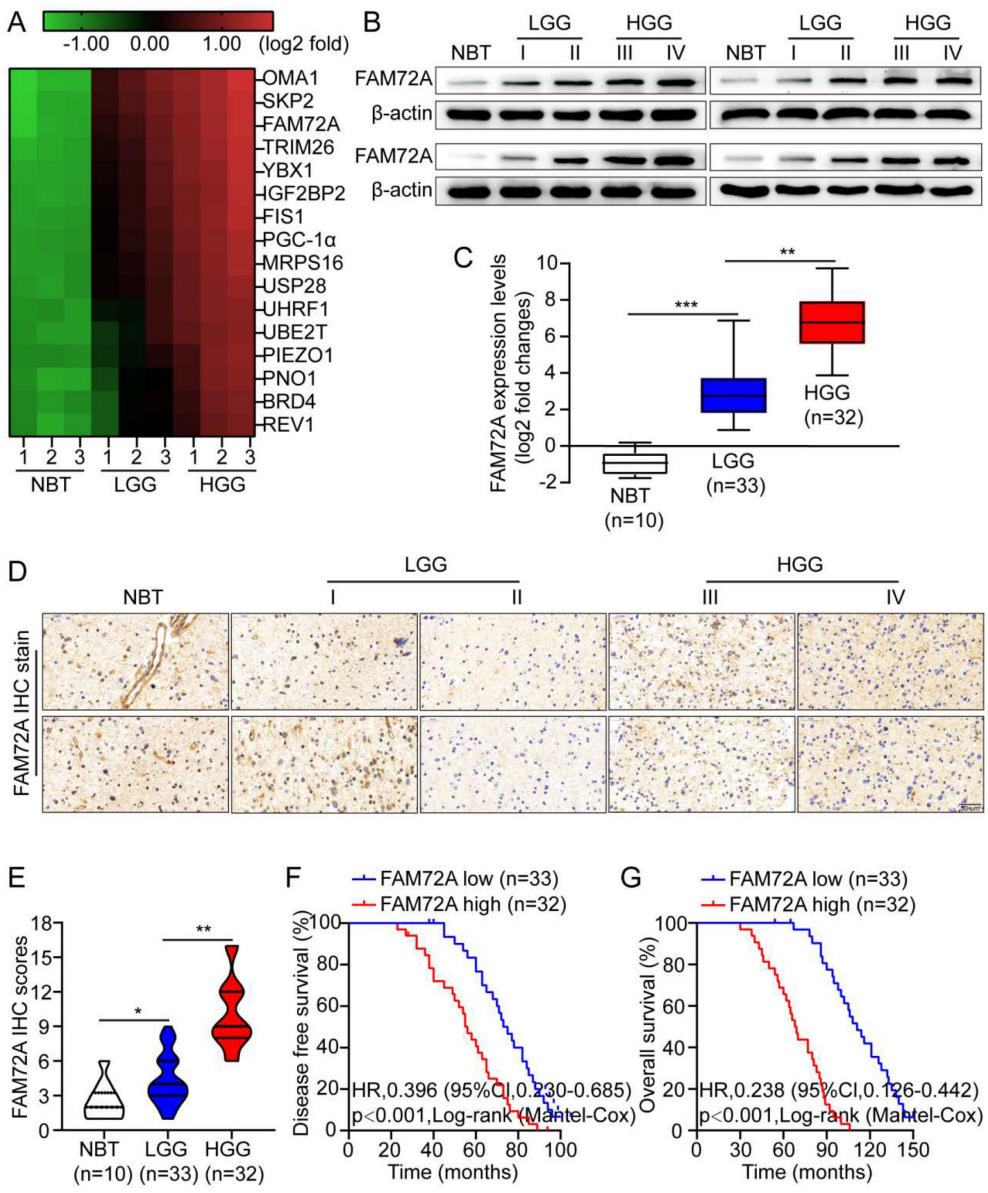
** Overexpression of FAM72A in human gliomas is associated with a poor prognosis. A** A hierarchical unsupervised clustering of proteins differentially expressed in normal brain tissue (NBT), low-grade gliomas (LGG) and high-grade gliomas (HGG). Based on a log2 transformation (fold changes > 5.0, p < 0.01), the pseudocolor represents NBT vs. LGG and LGG vs. HGG intensity. **B** In frozen lysates from 4 NBT and 16 glioma tissues, FAM72A protein expression levels were determined by Western blotting. **C** By RT-PCR, FAM72A mRNA expression levels were measured in four normal brain tissues (NBT) and 65 gliomas of different grades. Mean ± SD (n = 3), two-tailed t-test. **D** Representative immunohistochemical staining for FAM72A expression levels in NBT and different grades of gliomas. **E** Diagram showing the method used to quantify the mitochondrial-to-nuclear area ratio. **F-G** Kaplan-Meier analysis showed a negative correlation between poorer disease-free survival or overall survival rates and FAM72A mRNA expression levels. ANOVA (Dunnett's test) for multiple comparisons was performed as well as a two-tailed t-test to determine statistical significance. *P < 0.05, **P < 0.01 and ***P < 0.001.

To screen for key proteins responsible for glioma development, we performed next-generation sequencing (NGS) on three pairs of normal brain tissue (NBT), low-grade glioma (LGG) and high-grade glioma (HGG). The heatmap shows 16 proteins significantly upregulated in HGG relative to controls (Figure 1A). In the first step, Western blotting and quantitative RT-PCR were used to measure FAM72A levels in NBT and different grades of glioma tissues. Compared with NBT, FAM72A was often expressed at higher levels in tumor tissues, especially in HGG (Figures 1B-C). Next, IHC was used to measure the level of FAM72A in NBT as well as different grades of tumor tissues. As shown in Figure 1D, the IHC intensity of FAM72A was clearly greater in glioma tissue, particularly in HGG versus NBT. In addition, the quantitative analysis revealed that FAM72A protein levels were significantly higher in tumor tissue, especially in HGG than in NBT (Figure 1E). In addition, according to the Kaplan-Meier analysis, patients with high expression of FAM72A had a poorer prognosis (Figures 1F-G and Supplementary Figure 1A).

To assess the pathological and clinical predictive value of FAM72A, ROC analysis was conducted between FAM72A-based, WHO-based, and a combination of both to predict clinical outcomes. As measured by the area under the curve (AUC), the combination model (0.726) outperformed the WHO-based model alone (0.618). It seems that FAM72A combined with WHO stage could better predict clinical outcome than WHO stage alone (Supplementary Figures 1B-C). In addition, we examined the correlation between FAM72A mRNA levels and clinicopathological characteristics in 65 glioma specimens. As shown in Supplementary Figure 1C and Supplementary Table 1, the FAM72A mRNA levels were clearly associated with KPS score (P = 0.01) and recurrence (P = 0.024). The KPS predicts survival independently, which is consistent with the findings of previous studies 27. Furthermore, in univariate and multivariate Cox regression analyses, FAM72A mRNA levels were related to tumor grade and tumor recurrence (Supplementary Table 2). Overall, our data indicated that FAM72A may be a potential biomarker for glioma.

### Overexpression of FAM72A facilitates the progression of glioma

**Figure 2:**
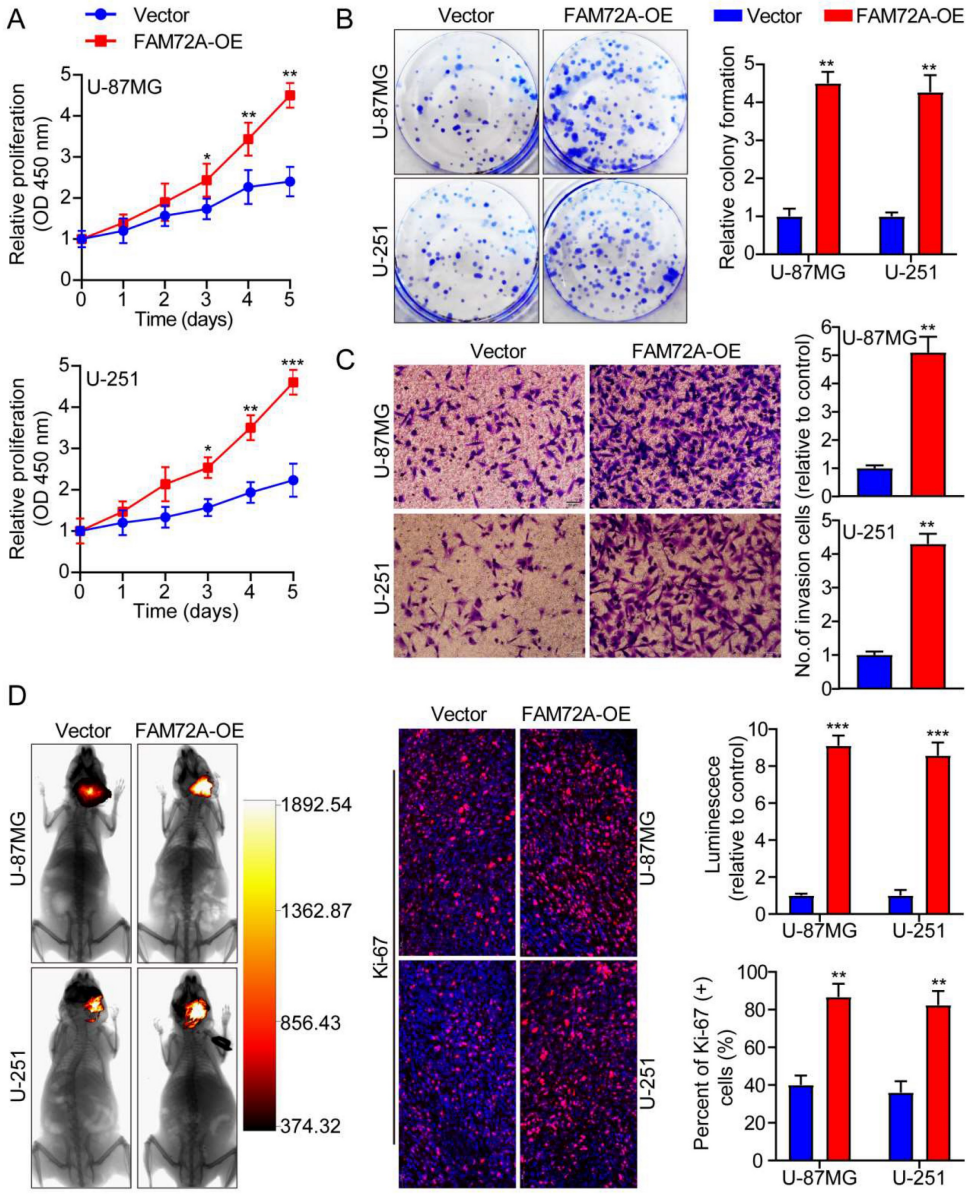
** Overexpression of FAM72A facilitates the progression of glioma. A** A comparison of the growth curves of Vector and FAM72A-OE by CCK-8 assay. **B** FAM72A overexpression facilitated colony formation and histogram quantification (panels). **C** Invasion assays showing that overexpression of FAM72A facilitates cell invasion. The numbers of invading cells are shown. Bars: 50 um. **D** Representative pictures of glioma *in situ* tumorigenesis and IF staining with Ki-67 are shown (n = 5 animals of 2 independent experiments). ANOVA (Dunnett's test) for multiple comparisons was performed as well as a two-tailed t-test to determine statistical significance. *P < 0.05, **P < 0.01 and ***P < 0.001.

We first measured the levels of FAM72A protein expression in two human brain gliocyte cell lines (HA and NHA) as well as five human brain glioma cell lines (A-172, LN-18, U-251, LN-229 and U-87MG). We selected U-87MG and U251 cells because they expressed FAM72A at the appropriate levels (Supplementary Figure 1D). We then overexpressed FAM72A in U-87MG and U-251 cells with lentiviruses targeting FAM72A, and FAM72A overexpression levels were measured by Western blotting (Supplementary Figure 1E). Subsequently, CCK-8, colony formation, and Transwell experiments were used to examine the effects of FAM72A on tumor cell growth, migration, and invasion.** The** data showed that FAM72A overexpression promoted tumor cell growth, migration, and invasion (Figures 2A-C and Supplementary Figure 2A). Nude mice were examined to determine whether FAM72A overexpression stimulated tumor growth *in vivo*. The results showed that, compared to the vector control, overexpression of FAM72A significantly increased tumor growth in glioma patients. In addition, Ki-67 IHC staining of tumor specimens in each group was performed. As shown in Figure 2D, the abovementioned findings were also confirmed. Taken together, these results demonstrated that the overexpression of FAM72A facilitated the progression of glioma.

### Knockdown of FAM72A inhibits the progression of glioma

**Figure 3:**
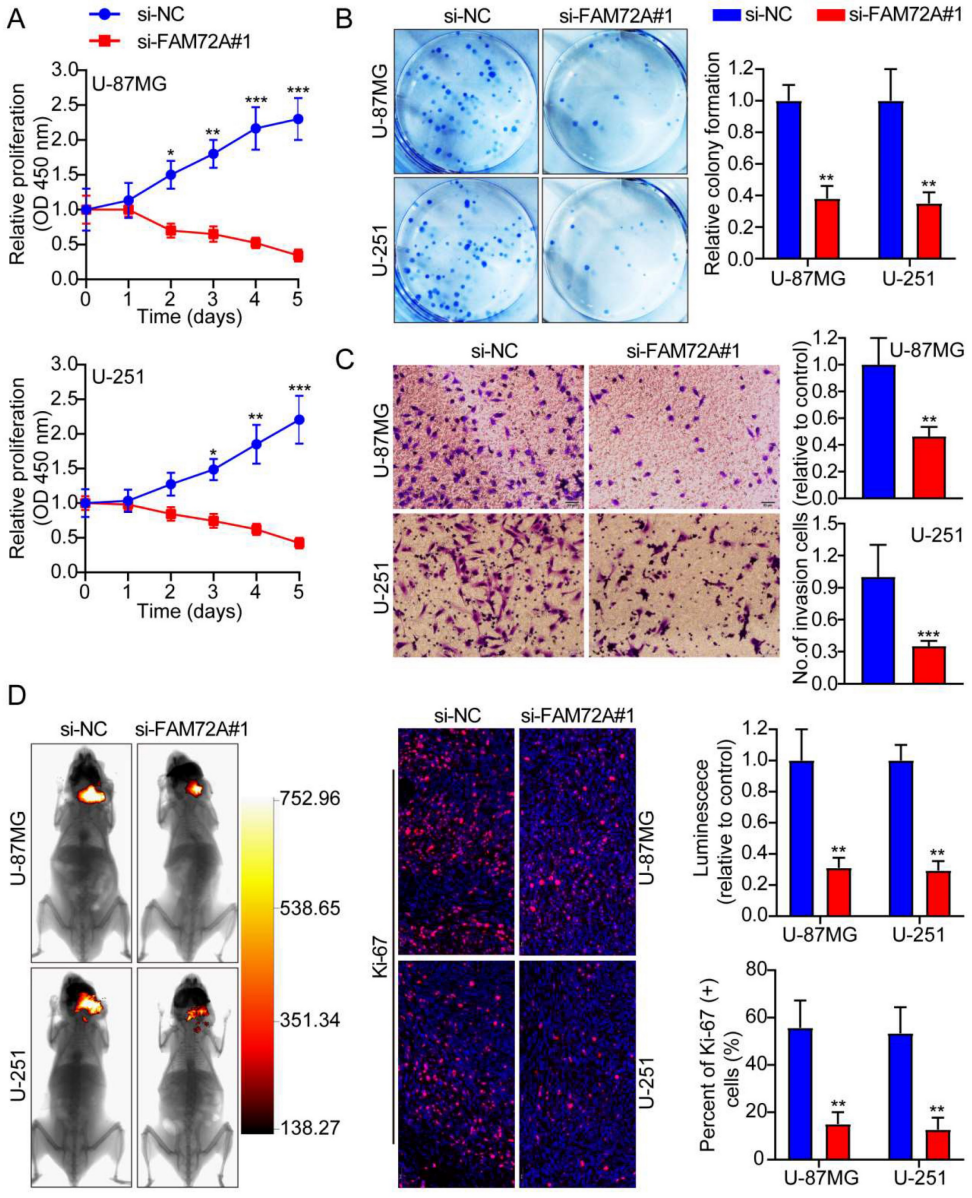
** Knockdown of FAM72A inhibits the progression of glioma. A** A comparison of the growth curves of si-NC and si-FAM72A#1 by CCK-8 assay. **B** FAM72A knockdown inhibited colony formation and histogram quantification (panels). **C** Invasion assays showing that knockdown of FAM72A suppressed cell invasion. The numbers of invading cells are shown. Bars: 50 um. **D** Representative pictures of glioma *in situ* tumorigenesis and IF staining with Ki-67 are shown (n = 5 animals of 2 independent experiments). An ANOVA (Dunnett's test) for multiple comparisons was performed as well as a two-tailed t-test to determine statistical significance. *P < 0.05, **P < 0.01 and ***P < 0.001.

After knocking down FAM72A in U-87MG and U-251 cells with lentiviruses targeting the gene, we measured its knockdown levels using Western blots (Supplementary Figure 1E). Then, we tested whether FAM72A affects tumor cell growth, migration, and invasion using CCK-8, colony formation, and Transwell experiments. The data indicated that FAM72A knockdown suppressed tumor cell growth, migration, and invasion (Figures 3A-C and Supplementary Figure 2B). The *in vivo* effects of FAM72A knockdown were investigated in naked mice. The data demonstrated that, compared to the vector control, knockdown of FAM72A clearly inhibited tumor growth in glioma patients. As described above, Ki-67 IHC staining of tumor specimens in each group was performed. As shown in Figure 3D, the abovementioned findings were also confirmed. Taken together, these data indicated that the knockdown of FAM72A suppressed the progression of glioma.

### FAM72A mediates glioma mitochondrial fission and fusion

**Figure 4:**
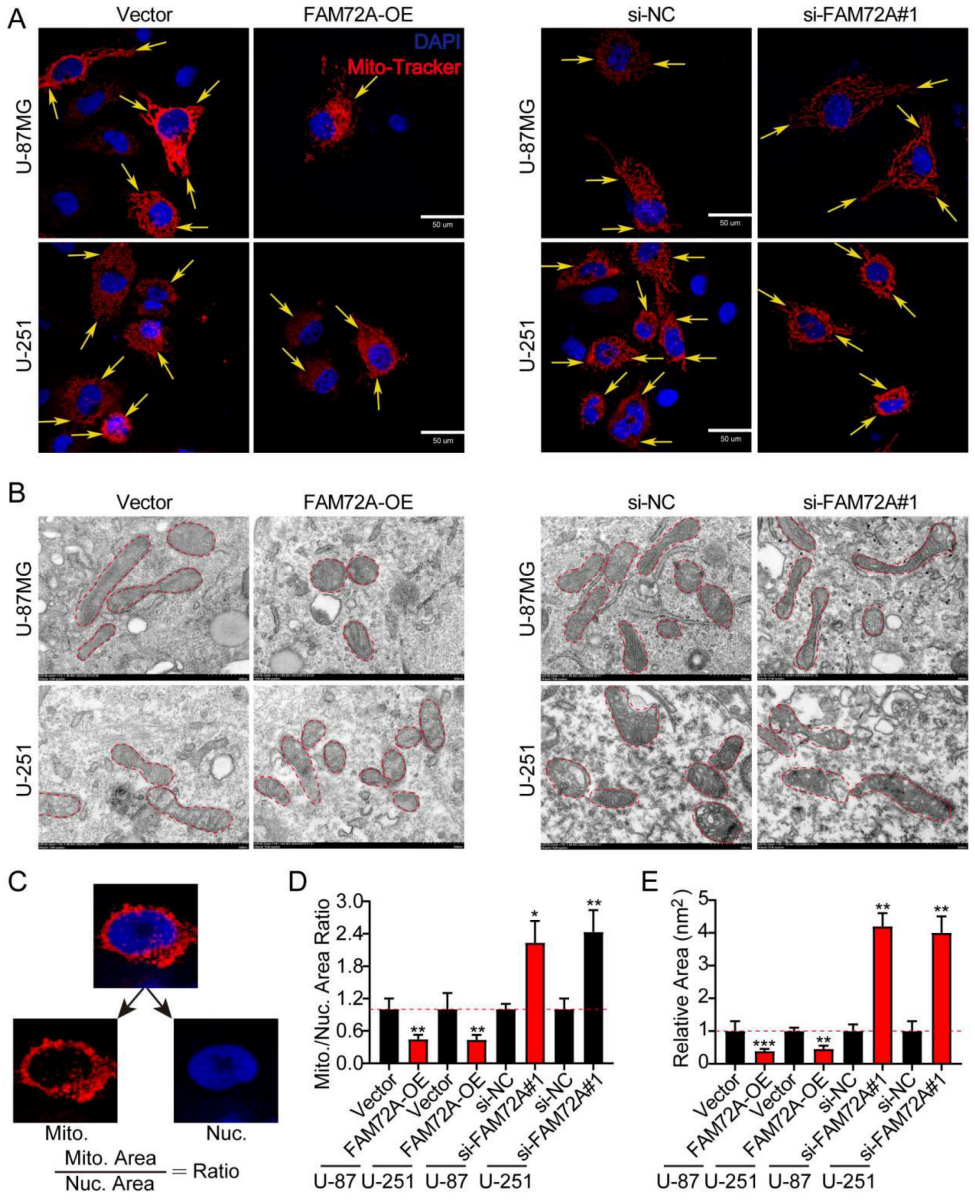
** FAM72A mediates glioma mitochondrial fission and fusion. A** Representative confocal images showing the morphology of the mitochondria in Vector vs. FAM72A-OE and si-NC vs. si-FAM72A#1. Yellow arrows indicate distinct mitochondrial morphology. **B** Representative TEM images showing the morphology of the mitochondria in Vector vs. FAM72A-OE and si-NC vs. si-FAM72A#1. Red dotted lines outline the mitochondrial shape. **C** Diagram showing the method used to quantify the mitochondrial-to-nuclear area ratio. **D** Mitochondrial to nuclear area ratio in Vector vs. FAM72A-OE and si-NC vs. si-FAM72A#1 in U-87MG and U-251 cells. Data are presented as the mean ± SD, two-tailed t-test. **E** Quantification of the mitochondrial cross-sectional area from the TEM images of U-87MG and U-251 cells. Data are presented as the mean ± SD, two-tailed t-test. *P < 0.05, **P < 0.01 and ***P < 0.001.

To further determine whether FAM72A regulates mitochondrial morphology, we knocked down and overexpressed FAM72A in both U-87MG and U-251 cells to verify its effect on mitochondrial morphology (Supplementary Figure 1D-E). Based on the role of FAM72A in mitochondrial dynamics, mitochondrial morphology in glioma was analyzed using confocal and transmission electron microscopy. DAPI and MitoTracker mitochondrial dyes were used for labeling for confocal microscopy analysis. Compared with that in the control groups, the mitochondrial-to-nuclear area ratio was significantly lower in the FAM72A overexpression groups, indicating that they contained fewer mitochondria. However, the FAM72A-depleted groups displayed the opposite results (Figures 4A-D). U-87MG and U-251 cells under different treatment conditions were examined by TEM to better understand mitochondrial morphology. Cross-sections of the mitochondria in the FAM72A overexpression groups consistently revealed a smaller area than those of the mitochondria in the control groups. However, the FAM72A-depleted groups displayed the opposite results (Figures 4B-E). In summary, these results indicated that FAM72A promoted the glioma mitochondrial fission.

### FAM72A promotes glioma progression via mitophagy

**Figure 5:**
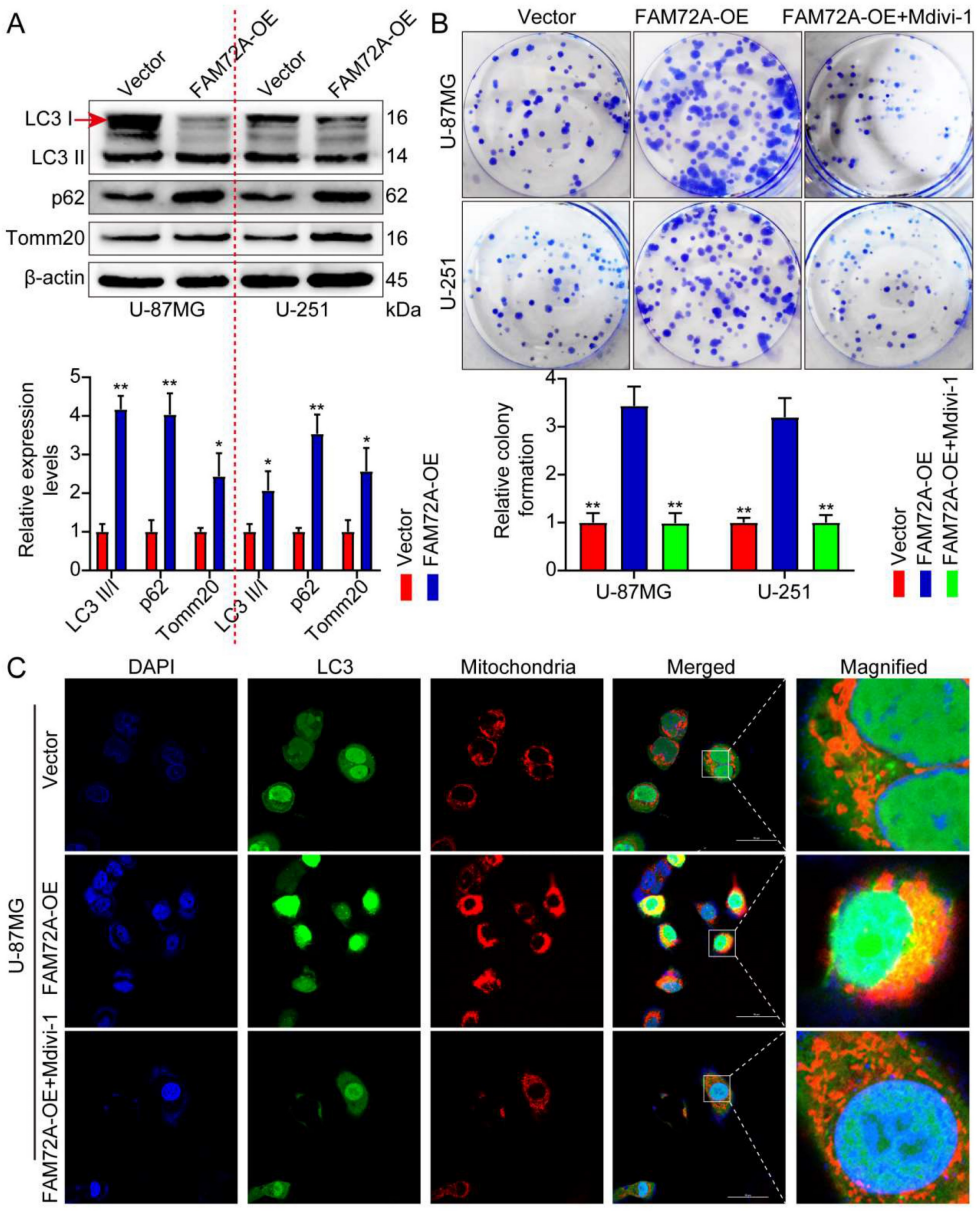
** FAM72A promotes glioma progression via mitophagy. A** WB to detect the expression levels of LC3 II/I, p62, and Tomm20 under the conditions of Vector and FAM72A-OE. The bar graph shows the results obtained after quantification (below). The experiment was repeated three times. Mean ± SD, two-tailed t-test. **B** Colony formation assay to detect the proliferation of U-87MG and U-251 cells under the conditions of Vector, FAM72A-OE, and FAM72A-OE + Mdivi-1. Histogram statistics of the relative colony formation of Vector, FAM72A-OE, and FAM72A-OE + Mdivi-1 (below). Each dot represents an individual cell. Mean ± SD, two-tailed t-test. **C** Representative confocal images showing the mitochondrial morphology and autophagy of U-87MG cells treated with Vector, FAM72A-OE, and FAM72A-OE + Mdivi-1. Red represents mitochondria; green represents mitophagy; yellow represents the fusion of red and green. *P < 0.05 and **P < 0.01.

We sought to determine whether FAM72A promotes glioma progression by promoting mitophagy. First, we overexpressed FAM72A. WB was performed to detect autophagy-related markers. FAM72A overexpression promoted autophagy (Figure 5A). The effect of FAM72A on glioma proliferation was determined using CCK-8, colony formation, and Transwell experiments. Subsequently, the cells were treated with the mitophagy inhibitor mitochondrial division inhibitor 1 (Mdivi-1) while overexpressing FAM72A. Mdivi-1 is a selective and cell-penetrating inhibitor of mitochondrial division that inhibits dynamin-related GTPase (DRP1) and dynamin I (Dnm1), with an IC50 of 1-10 μM. Mdivi-1 reduces mitophagy and increases apoptosis 28. The overexpression of FAM72A promoted tumor cell growth, migration, and invasion. However, the addition of Mdivi-1 weakened the ability of tumor cells to overexpress FAM72A (Figure 5B, Supplementary Figures 2C and 3A-B). MitoTracker was used to label the mitochondria, while LC3 was used to label autophagy; the colocalization of the two was then used to measure mitophagy under different treatment conditions. Compared with the control group, FAM72A overexpression enhanced autophagy, and Mdivi-1 attenuated the enhanced autophagy of tumor cells caused by the overexpression of FAM72A (Figure 5C, Supplementary Figures 2D-E and 3C). Taken together, these data indicated that FAM72A promoted glioma progression via mitophagy.

### FAM72A promotes mitophagy and maintains Pink1 stability through Pink1/Parkin

**Figure 6:**
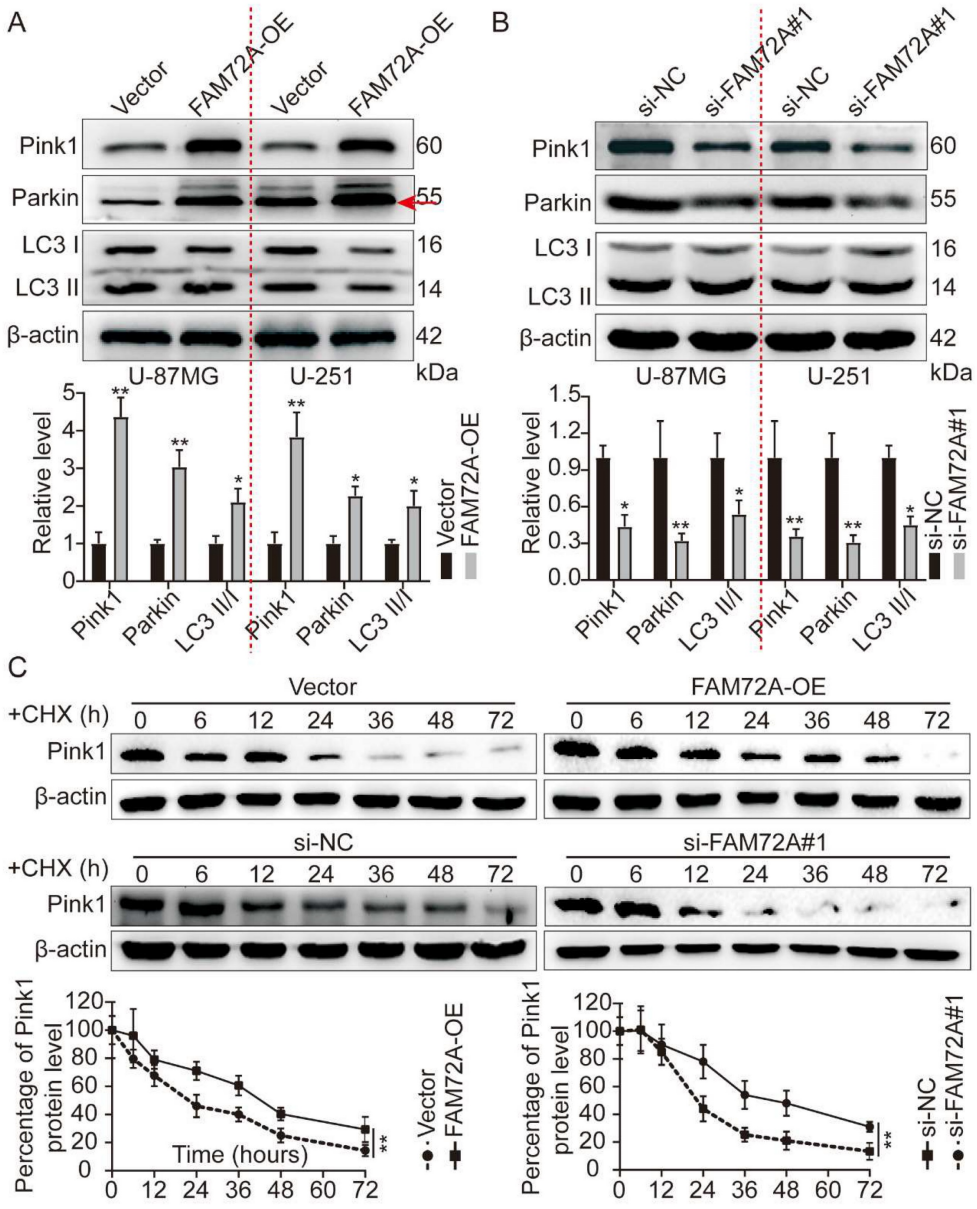
** FAM72A promotes mitophagy and maintains Pink1 stability through Pink1/Parkin. A** WB to detect the expression levels of Pink1, Parkin, and LC3 II/I under the conditions of Vector and FAM72A-OE. The bar graph shows the results obtained after quantification (below). **B** WB to detect the expression levels of Pink1, Parkin, and LC3 II/I under the conditions of si-NC and si-FAM72A#1 conditions. The bar graph shows the results obtained after quantification (below). **C** WB to detect the expression levels of Pink1 under the conditions of Vector and FAM72A-OE or si-NC and si-FAM72A#1 by CHX assays. The bar graph shows the results obtained after quantification (below). All experiments were repeated three times. Mean ± SD, two-tailed t-test. (*P < 0.05 and **P < 0.01).

In most cells, mitochondrial oxidative phosphorylation provides energy. Thus, it is essential to degrade damaged mitochondria efficiently and replace them in a timely manner to maintain mitochondria's normal physiological function 2, 4, 5. In a recent study, Pink1 and Parkin proteins related to autosomal recessive juvenile Parkinson's disease were shown to influence mitochondrial fusion and fission dynamics jointly. By promoting autophagy, damaged mitochondria are selectively degraded. Known as Pink1/Parkin-mediated mitophagy, Pink1 or Parkin mutation inhibits mitophagy, one of the important mechanisms of Parkinson's disease 11, 12, 29. First, we overexpressed and knocked down FAM72A and measured the levels of Pink1/Parkin by WB, as well as mitophagy markers. FAM72A overexpression increased Pink1, Parkin, and LC3 II/I expression levels (Figure 6A). In contrast, knockdown of FAM72A inhibited Pink1 and Parkin expression levels as well as LC3 II/I (Figure 6B). Next, we investigated how FAM72A stimulates Pink1 expression. Pink1 expression was detected under different treatment conditions using WB and CHX assays to inhibit protein synthesis. In comparison to the control, FAM72A overexpression prevented Pink1 degradation, whereas FAM72A knockdown led to Pink1 degradation (Figure 6C). Through Pink1/Parkin, FAM72A promotes mitophagy and maintains the stability of Pink1.

### FAM72A promotes glioma immune escape by upregulating PD-L1 expression

**Figure 7:**
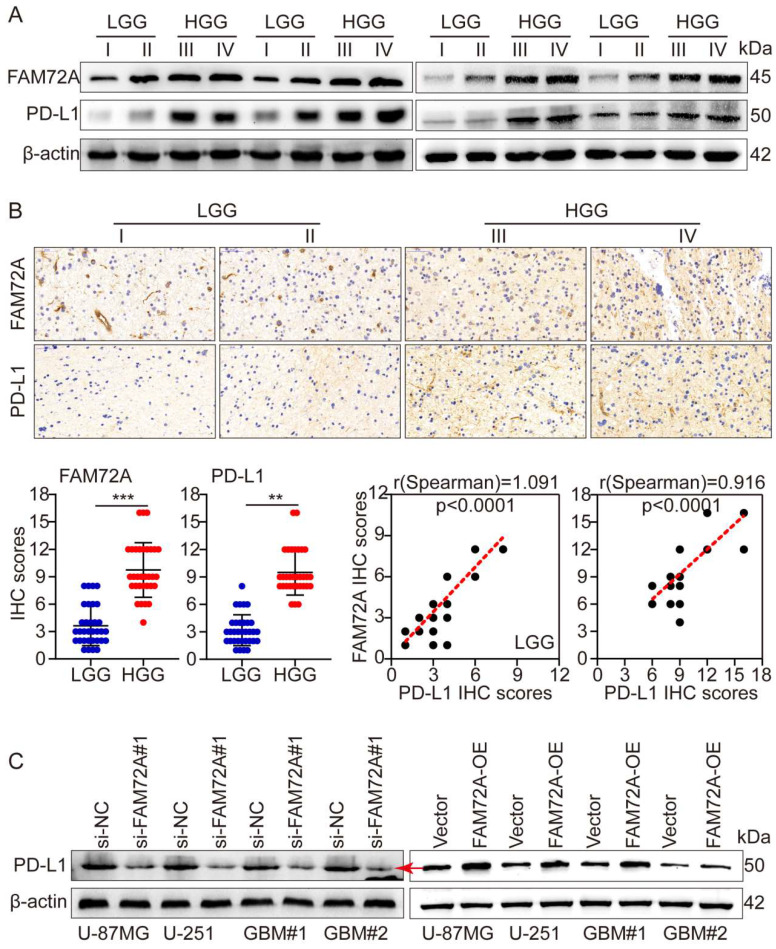
** FAM72A promotes glioma immune escape by upregulating PD-L1 expression. A** WB to detect the expression levels of FAM72A and PD-L1 in LGG and HGG. **B** IHC to detect the expression levels of FAM72A and PD-L1 in LGG and HGG. The median FAM72A and PD-L1 expression levels were used as cutoff values. The figure below shows the IHC scores of FAM72A and PD-L1, and the correlation analysis between the two scores. **C** WB to detect the expression levels of PD-L1 under the conditions of si-NC and si- FAM72A#1, or Vector and FAM72A-OE. All experiments were repeated three times. Mean ± SD, two-tailed t-test. (**P < 0.01 and ***P < 0.001).

A major breakthrough in tumor therapy in the past decade has been immunotherapy, especially with the introduction of PD1/PD-L1 immune checkpoint inhibitors. Although immunotherapy has vast prospects in hematological and a few solid tumors, its efficacy in most solid tumors, such as gliomas, remains extremely limited. This result is obtained primarily due to the tumor microenvironment inhibiting the function of immune cells. To further investigate the mechanism of glioma immune escape, WB and IHC were used to detect PD-L1 levels in different grades of gliomas. Based on the grade of the tumor, both FAM72A and PD-L1 expression increased. Additionally, WB and IHC showed that FAM72A and PD-L1 levels were higher in HGG samples than in LGG samples. According to the IHC scores, FAM72A and PD-L1 were positively correlated (Figures 7A-B). Following knockdown and overexpression of FAM72A, WB was used to detect PD-L1 expression levels. FAM72A knockdown suppressed PD-L1 expression, whereas FAM72A overexpression increased PD-L1 expression (Figure 7C).

## Discussion

Malignant brain tumors called gliomas are the most common type, and they remain incurable despite advances in medicine 30. The prognosis for patients diagnosed with gliomas is terrible, with a median survival time of 12-15 months 31, 32. In addition to the rapid proliferation, invasive nature, genetic heterogeneity, and treatment resistance of gliomas, a lack of understanding of the molecular mechanisms that control the progression of glioma also contributes to the low survival rate of patients with the disease 33, 34. Therefore, new glioma biomarkers with strong prognostic value are urgently needed.

Mitochondrial health involves careful regulation of the pathways involved in determining mitochondrial size, biogenesis, and degradation 35, 36.

Mitochondrial size changes dynamically during mitochondrial fusion (expansion) and fission (contraction). Fission-related mitochondrial dysfunction is eliminated by mitophagy. Activation of Pink1/Parkin after mitochondrial depolarization is one of the most studied mitophagy pathways. As it passes through the inner mitochondrial membrane, Pink1 is cleaved by PARL and targeted for degradation. In humans, Parkin contains 465 amino acids and is an E3 ubiquitin ligase that attaches death tags to useless proteins for proteases to recognize 10, 12. A key part of the autophagy pathway involves the accumulation of Pink1 in damaged mitochondria, where it recruits the E3 ubiquitin ligase Parkin to attack the mitochondria and initiate autophagy 9. Several pathological conditions, including neurodegeneration 37, muscular dystrophy 38, cardiovascular disease 39, metabolic disorders 40, liver disease 41, and cancer 42, are caused by excessive or insufficient mitophagy. Damaged mitochondria accumulate in cells due to defective mitophagy, which leads to cell death. Early onset Parkinson's disease is associated with mutations in the Pink1 and Parkin genes 10, 37. Loss of mitophagy and mitochondrial dysfunction are associated with these diseases. Consistent with previous reports, we found that FAM72A-mediated mitophagy can promote glioma progression to maintain mitochondrial homeostasis.

In 2018, the Nobel Prize in Physiology or Medicine was awarded for research on immune checkpoints, represented by PD-1 and CTLA-4. A major inhibitory molecule of T lymphocytes, PD-1 was discovered in mouse T lymphocytes with activated T cells receptors (TCRs). Tumor-infiltrating lymphocytes have been found to express high levels of PD-1 in a variety of cancers 43. As a natural ligand for PD-1, there are two classes: PD-L1 and PD-L2. PD-L1 can be induced by many cytokines and is widely expressed by various types of cells, such as tumor cells, fibroblasts, and macrophages 44. The PD-1 receptor transmits inhibitory signals upon binding to its ligand, decreases TCR signaling pathway phosphorylation, decreases TCR activation downstream of the receptor, and protects tumor cells from CD8+ T lymphocyte cracking by inhibiting T lymphocyte proliferation and function 45. The ligand of PD-1 promotes tumor cell escape from the immune system if it is expressed on the surface of tumor cells. Anti-PD-1 or PD-L1 mAbs block binding and restore the antitumor activity of T lymphocytes 46.

In addition to PD-L1, PD-L2 interacts with PD-1 to transmit inhibitory immune response signals. However, because PD-L2 expression is relatively rare in tumor cells, PD-L2 plays a lesser role in tumor development 47. Herein, FAM72A was demonstrated to promote immune evasion of glioma by upregulating PD-L1 expression.

In this study, we report the clinical relevance and function of FAM72A in gliomas. FAM72A was frequently upregulated in glioma tissues, and knockdown of FAM72A suppressed tumor progression. However, FAM72A overexpression promoted tumor progression. Mechanistically, we found that FAM72A promoted glioma progression by regulating mitophagy through the Pink1/Parkin signaling pathway. In addition, FAM72A promoted mitophagy and maintained Pink1 stability through the Pink1/Parkin signaling pathway. Finally, FAM72A promoted tumor immune escape by upregulating PD-L1 expression.

In conclusion, our study highlighted the importance of FAM72A in modulating glioma, thereby promoting glioma development. Thus, FAM72A may be a useful biomarker for identifying and treating patients with glioma. In particular, a novel clinical treatment strategy is provided for patients with gliomas.

## Supplementary Material

Supplementary methods, figures and tables.
