# FAM72A promotes glioma progression by regulating mitophagy through the Pink1/Parkin signaling pathway: Erratum

**DOI:** 10.7150/jca.93189

**Published:** 2024-02-01

**Authors:** Yibin Zeng, Cui Xiong, Nan Tang, Siqi Wang, Zhiyong Xiong, Tao Liang, Qiangping Wang, Menglong Li, Junjun Li

**Affiliations:** 1 Department of Neurosurgery, Union Hospital, Tongji Medical College, Huazhong University of Science and Technology, 1277 Jiefang Avenue, Wuhan, Hubei, 430022, China.; 2 Department of Endocrinology, Union Hospital, Tongji Medical College, Huazhong University of Science and Technology, 1277 Jiefang Avenue, Wuhan, Hubei, 430022, China.; 3 Department of Radiology, Union Hospital, Tongji Medical College, Huazhong University of Science and Technology, 1277 Jiefang Avenue, Wuhan, Hubei, 430022, China.; 4 Department of Clinical Laboratory, Union Hospital, Tongji Medical College, Huazhong University of Science and Technology, 1277 Jiefang Avenue, Wuhan, Hubei, 430022, China.; 5 Department of Neurosurgery, Nanshi Hospital of Nanyang, Henan University, No. 988, Zhongzhou West Road, Nanyang City, 442000, China.

In the original version of our article, there were three errors in the Supplementary Table 3, Supplementary Table 4 and Supplementary Table 5. Specifically, the siRNA sequence information in Supplementary Table 3 was wrong, the correct siRNA sequence is provided below: siFAM72A-1: GTACAGATGAAGATGTGTTAA and siFAM72A-2: GCTCAGCATGATGTTAGATAA.

The antibody number in Supplementary Table 4 was wrong, the correct numbers were nbp2-59790(Novus, Colorado, USA) and orb863765 (Cambridge, United Kingdom). In addition, the FAM72A primers (human) in Supplementary Table 5 was missed, in fact, we also detected Fam72a gene expression in mouse brain tumors and normal brain tissues in the previous experiment (results not shown in the article), but only mouse primers were provided in the supplementary materials, and we made the following supplements: human FAM72A qPCR primers: F: GGAATGAAGGCTGTTTTGCTGGC; R: AACATGCGATGTCCTTCAGTTTAC. The mouse Gapdh qPCR primers are as follows: F: AGGTCGGTGTGAACGGATTTG; R: GGGGTCGTTGATGGCAACA.

We are truly sorry for these errors. This correction will not affect the results and conclusions. The authors apologize for any inconvenience this may have caused.

