# Supplementary material for: FAM72A promotes glioma progression by regulating mitophagy through the Pink1/Parkin signaling pathway
Source: J Cancer. 2023 Apr 2;14(6):903–15. doi: 10.7150/jca.82949 (PMC10158506; doi:10.7150/jca.82949)
Supplement: Supplementary file 1 — Supplementary methods, figures and tables. [file jcav14p0903s1.pdf]

FAM72A promotes glioma progression by regulating mitophagy through the Pink1/Parkin signaling pathway

Yibin Zeng[1], Cui Xiong[2], Nan Tang[1], Siqi Wang[3], Zhiyong Xiong[1], Tao Liang[4], Qiangping Wang[1], Menglong Li[5*], Junjun Li[1*]

Supplementary Methods

Clinical samples

Informed consent was provided by all patients, and the Ethics Committee of Wuhan Union Hospital (Wuhan, China) (No. 2020-002) approved all aspects of this study. Each specimen was divided into two samples for: (i) formalin fixation and paraffin embedding, (ii) protein and RNA extraction.

Cell culture and treatment

HA, NHA, A-172, LN-18, U-251, LN-229 and U-87MG cells were obtained from the American Type Culture Collection (Manassas, VA, USA) cultured in Dulbecco's modified Eagle's medium (Gibco, Waltham, MA, USA) supplemented with 10% fetal bovine serum (Gibco), glutamine (Gibco), and 1% penicillin-streptomycin (Gibco) in 5% CO2 at 37 °C. All cells cultures were established as described previously [1-2]. Short tandem repeat (STR) analysis was done on all cells and they were regularly tested for mycoplasma contamination. Mdivi-1 was obtained from Selleck (S7162, Selleck Chemicals, Shanghai, China).

Plasmids, small interfering RNA (siRNA), and transfection

U-87MG and U-251 cells were transfected with siRNAs targeting FAM72A using Lipofectamine 3000 (Invitrogen, Waltham, MA, USA) as per the manufacturer's instructions. The full-length cDNA of FAM72A were cloned from template-strand cDNA by PCR. FAM72A vectors were constructed by cloning full-length cDNA into pcDNA3.1 vectors (Sigma-Aldrich, St Louis, MO, USA).

Real-time quantitative RT-PCR (qRT-PCR)

Total RNA was extracted from tissue samples using TRIzol (Invitrogen). cDNA was synthesized using the HiScript III 1st Strand cDNA Synthesis Kit (R312-01; Vazyme,

Nanjing, China) according to the manufacturer’s instructions. RT-qPCR analysis was performed on a 7500 Fast Real-Time PCR system (Applied Biosystems, Foster City, CA, USA) using the AceQ qPCR SYBR Green Master Mix (Q111-C1; Vazyme). The primers used in the present study were chemically synthesized by GeneCreate (Wuhan, China).

Bioinformatic analysis

The fragments per kilobase of transcript per million mapped reads (FPKM) transcriptome RNA-sequencing data and clinical features of patients with glioma were extracted from The Cancer Genome Atlas (TCGA) (https://cancergenome.nih.gov/). FPKM transcriptome RNA-sequencing data of normal brain tissue were downloaded from the Genotype Tissue Expression (GTEx) database [3]. Survival curves were constructed using the Kaplan–Meier method and differences between groups were compared using the log-rank test. The results of the independent sample *t*-tests comparing the two groups were expressed as *t*-test values. P value < 0.05 was considered significant (ns: p 0.05, *: $p \leq 0.05$, **: $p \leq 0.01$, ***: $p \leq 0.001$). Correlation analyses were performed using Pearson correlation.

Supplementary Tables 1: Correlation of the expression levels of FAM72A in glioma tissues with clinicopathologic features.

**Characteristics**	**No. of cases (%)**	**FAM72A**		**P-value**
		**Low**	**High**	
**Age (y)**				
<50	35 (53.8%)	15	20	0.16
≥50	30 (46.2%)	18	12	
**Gender**				
Male	32 (49.2%)	17	15	0.69
Female	33 (50.8%)	16	17	
**Tumor size (cm)**				

<2	37 (56.9%)	25	12	0.002**
≥2	28 (43.1%)	8	20	
**Tumor location**				
Supratentorial	31 (47.7%)	16	15	0.88
Subtentorial	34 (52.3%)	17	17	
**Karnofsky performance scale**				
<90	30 (46.2%)	10	20	0.01**
≥90	35 (53.8%)	23	12	
**WHO grade**				
I	5 (7.7%)	4	1	0.0002***
II	20 (30.8%)	16	4	
III	16 (24.6%)	9	7	
IV	24 (36.9%)	4	20	
**Tumor recurrence**				
No	30 (60.0%)	20	10	0.024*
Yes	34# (40.0%)	13	21#	

Partial data not available; statistics based on available data. *<0.05, **<0.01 and ***<0.001. FAM72A high expression: score 8-16; low expression: score 0-7.

Supplementary Tables 2: Univariate and multivariate for clinicopathological features associated with various prognostic parameters of 65 glioma patients by Cox-regression analysis.

Variables	Univariate analysis		Multivariate analysis	
	HR (95%CI)	P-value	HR (95%CI)	P-value
Age (≥50 vs <50)	1.285 (1.075-2.193)	0.463	1.571 (1.219-3.281)	0.375
Gender (Male vs Female)	1.732 (1.254-4.258)	0.537	1.583 (1.176-3.638)	0.426
Tumor size (≥2 vs <2)	1.758 (1.386-3.537)	0.035*	1.582 (1.125-3.258)	0.085
Tumor location (Sup vs Sub)	1.639 (1.235-3.682)	0.542	1.384 (1.058-2.385)	0.856

KPS (≥90 vs <90)	2.537 (1.462-4.386)	0.025*	1.793 (1.354-3.783)	0.038*
WHO grade (HGG vs LGG)	0.652 (0.254-1.728)	0.005**	0.854 (0.357-2.279)	0.012*
Tumor recurrence (Yes vs No)	2.693 (1.585-5.572)	0.032*	3.284 (1.872-6.693)	0.038*
FAM72A (Low vs High)	2.563 (1.632-4.842)	0.012*	2.627 (1.365-5.361)	0.025*

Supplementary Tables 3:

**Gene**	**Primer**	**Sequence(5′-3′)**
si-FAM72A#1	forward	GCAAGTCAACAGGAAGGGT
	reverse	TACFFAFFAAFFTAACAG
si-FAM72A#2	forward	TCCAGGTAGTGTAAGCCCTCA
	reverse	TCCTGTTCACTCCTCAGATCG

Supplementary Tables 4:

Name	Description
Anti-FAM72A antibody	ab121364 (Abcam, Cambridge, USA)
Anti-Pink1 antibody	ab216144 (Abcam, Cambridge, USA)
Anti-Parkin antibody	ab77924 (Abcam, Cambridge, USA)
Anti-LC3A/B antibody	ab128025 (Abcam, Cambridge, USA)
Anti-PD-L1 antibody	66248-1-lg (Proteintech, Wuhan, China)
Anti-p62 antibody	ab109012 (Abcam, Cambridge, USA)
Anti-TOMM20 antibody	ab186735 (Abcam, Cambridge, USA)
Anti-β-actin antibody	#4970 (Cell Signaling Technology, Beverly, MA, USA)

Supplementary Tables 5:

Gene	Primer	Sequence (5′-3′)
FAM72A	forward	TTTCAAAGACCGATGCGTATCC
	reverse	CTATGTCAGTATCAGCCAGCAAA
GAPDH	forward	GAGTCAACGGATTTGGTCGT
	reverse	TTGATTTTGGAGGGATCTCG

Supplementary Figure Legends

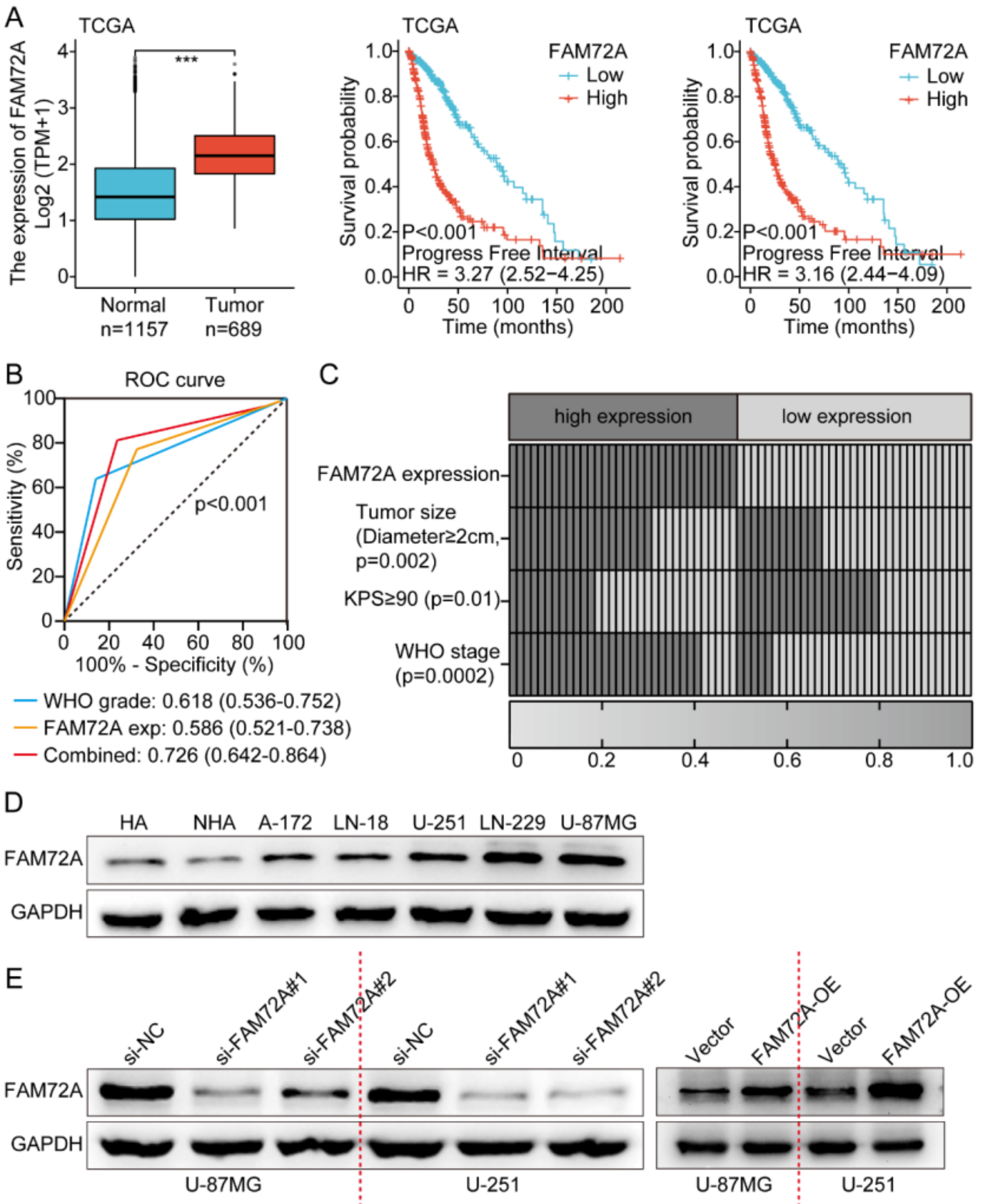

Fig. S1 **A** The results of the TCGA database showed that FAM72A was highly expressed in tumor tissues, and the expression level was inversely proportional to the prognosis of patients. **B** ROC analysis of FAM72A-based, WHO-based and the combination model in predicting clinical outcome. **C** The heatmap illustrates the association of different clinical characters with FAM72A high and low-expression tumors. **D** WB to detect the expression levels of FAM72A between HA, NHA, A-172, LN-18, U-251, LN-229 and U-87MG cells. **E** Validation of knockdown and overexpression efficiency of FAM72A by WB. Data shown are mean±SD (n = 3). (***P < 0.001).

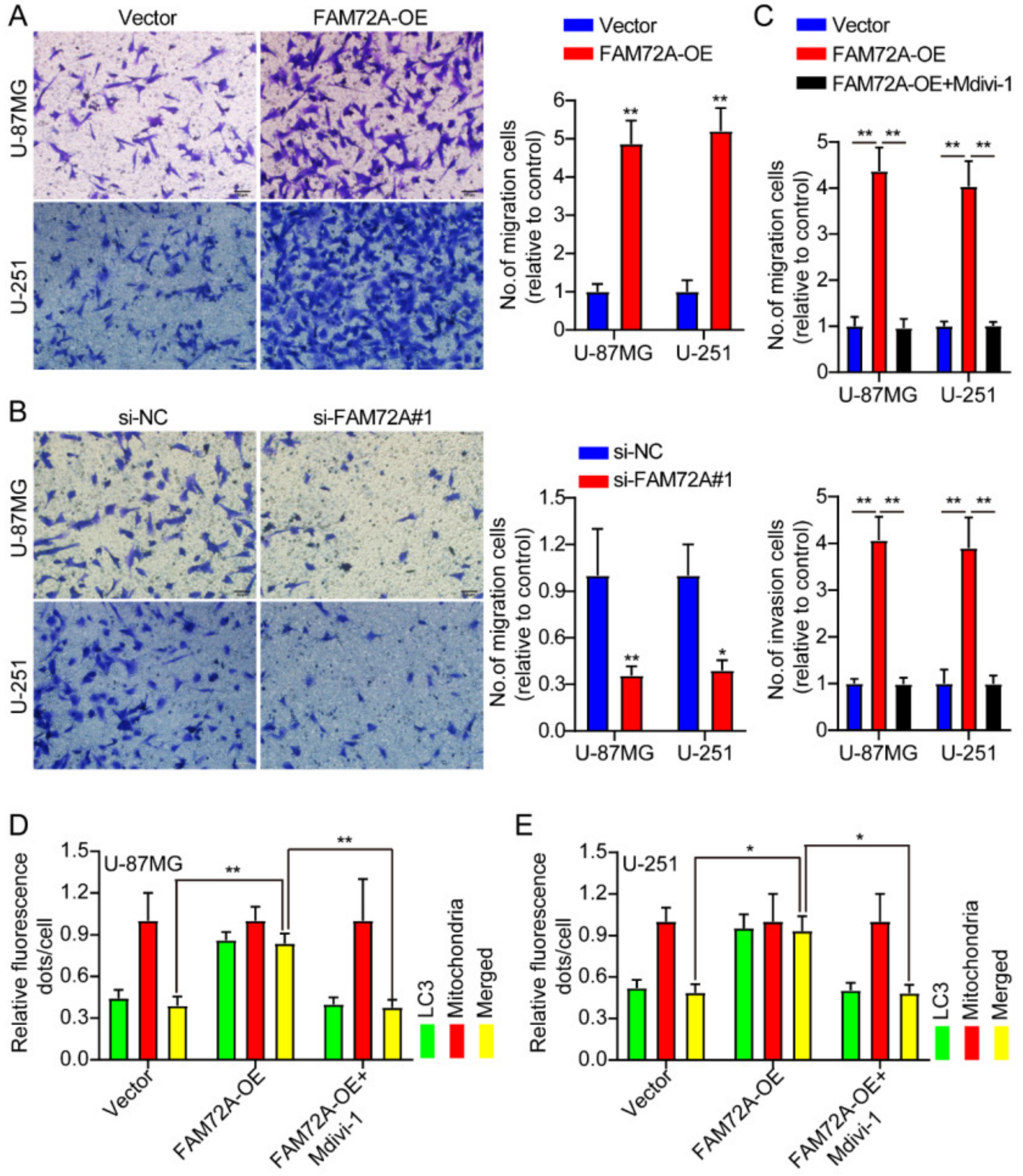

Fig. S2 A Migration assays showing that overexpression of FAM72A promote cell migration. The numbers of migrating cells are shown. Bars: 50um. **B** Migration assays showing that knockdown of FAM72A inhibit cell migration. The numbers of migrating cells are shown. Bars: 50um. **C** Histogram statistics of the relative migrating and invasion cells of Vector, FAM72A-OE, and FAM72A-OE + Mdivi-1. **D-E** Relative fluorescence spot numbers per Vector, FAM72A-OE, and FAM72A-OE + Mdivi-1 cells. A total of 100 cells were counted for each analysis. All experiments were repeated

three times. Mean ± SD, two-tailed t-test. (*P < 0.05 and **P < 0.01).

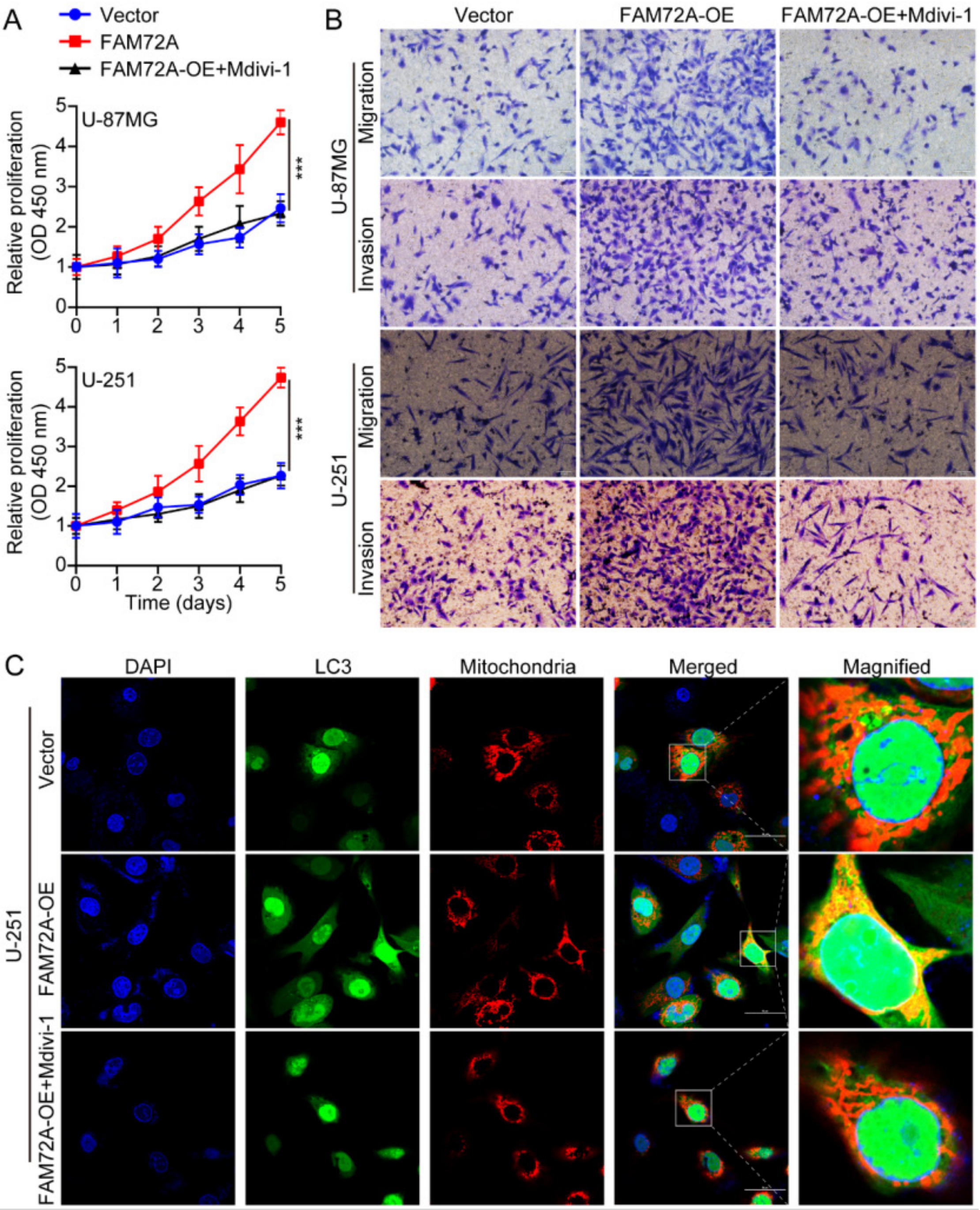

Fig. S3

A A comparison of the growth curves of Vector, FAM72A-OE, and FAM72A-OE + Mdivi-1 group by CCK-8 assay.

B Representative images showing the migration and invasion of U-87MG and U-251 cells on day 2 between Vector, FAM72A-OE, and FAM72A-OE + Mdivi-1 groups.

c Representative confocal images showing the mitochondrial morphology and

autophagy of U-251 cells treated with Vector, FAM72A-OE, and FAM72A-OE + Mdivi-1. Red represents mitochondria; green represents mitophagy; yellow represents the fusion of red and green. ***P < 0.001.

References

1. Li J, Liao T, Liu H, Yuan H, Ouyang T, Wang J, Chai S, Li J, Chen J, Li X, et al: Hypoxic Glioma Stem Cell-Derived Exosomes Containing Linc01060 Promote Progression of Glioma by Regulating the MZF1/c-Myc/HIF1alpha Axis. *Cancer Res* 2021, 81:114-128.

2. Li J, Yuan H, Xu H, Zhao H, Xiong N: Hypoxic Cancer-Secreted Exosomal miR-182-5p Promotes Glioblastoma Angiogenesis by Targeting Kruppel-like Factor 2 and 4. *Mol Cancer Res* 2020, 18:1218-1231.

3 Consortium GT: The Genotype-Tissue Expression (GTEx) project. *Nat Genet* 2013, 45:580-585.
